# The presence of membrane bound CD99 ligands on leukocyte surface

**DOI:** 10.1186/s13104-020-05347-0

**Published:** 2020-10-22

**Authors:** Nuchjira Takheaw, Supansa Pata, Witida Laopajon, Sittiruk Roytrakul, Watchara Kasinrerk

**Affiliations:** 1 Division of Clinical Immunology, Department of Medical Technology, Faculty of Associated Medical Sciences, Chiang Mai University, Chiang Mai, Thailand; 2 Biomedical Technology Research Center, National Center for Genetic Engineering and Biotechnology, National Science and Technology Development Agency at the Faculty of Associated Medical Sciences, Chiang Mai University, Chiang Mai, Thailand; 3 Functional Ingredients and Food Innovation Research Group, National Center for Genetic Engineering and Biotechnology, National Science and Technology Development Agency, Pathumtani, Thailand

**Keywords:** CD99 ligand, CD99, Recombinant CD99, LC–MS/MS, Leukocyte

## Abstract

**Objective:**

CD99, a leukocyte surface molecule, reportedly plays an important role in several cellular processes. However, the role of CD99 in T cell regulation remains unclear, as the CD99 ligand associated with T-cell regulation has not yet been identified. Our previous study showed that recombinant CD99 bound to CD99 ligands was expressed on monocytes, NK cells and dendritic cells. This interaction regulates the expression of IL-6 and TNF-α in CD3 + T cells following T cell activation. In the present study, we confirmed the presence of CD99 ligands in immune cells.

**Results:**

A recombinant CD99-human IgG fusion protein, CD99HIgG, was produced and used to search for CD99 ligand expression in various hematopoietic cell lines. Among several cell lines, THP-1 monocytic cell line showed strong positive reaction for CD99HIgG, and CD99 and CD99 ligand complexes were pulled-down using a DTSSP cross-linker. The study demonstrated the presence of the membrane bound CD99 ligand, and CD99 ligand candidates were identified via LC–MS/MS. These results may be useful to further identify the CD99 ligands, and to fully comprehend the role of CD99 in immunoregulation.

## Introduction

CD99 is a heavily O-linked type I transmembrane glycoprotein expressed on both hematopoietic and non-hematopoietic cells [1–3]. Up-regulation of CD99 expression has been demonstrated in activated and memory T cells [4, 5]. CD99 reportedly plays a key role in several cellular processes including cell adhesion, migration, differentiation and cell death [6, 7]. Furthermore, it has been proposed that CD99 may function as either an activating or inhibitory receptor in T cell regulation. Upon T cell activation, anti-CD99 monoclonal antibodies (mAbs) induced T cell signalling and functions was demonstrated [8–10]. In contrast, the inhibition of T cell responses using a distinct clone of anti-CD99 mAb has also been reported [11, 12]. In most studies of the role of CD99 in T cell regulation, mAbs were used to mimic its ligands, and different mAbs resulted in diverse functional CD99 outcomes [2, 13]. Hence, the role of CD99 in immunoregulation remains controversial. This could be attributed to the fact that the CD99 ligand has not yet been identified.

Recently, we demonstrated that putative CD99 ligands were expressed on the surfaces of monocytes, NK cells and dendritic cells, but not on those of B and T cells [14]. Recombinant CD99 protein enhanced the upregulation of IL-6 and TNF-α expression by T cells, monocytes and NK cells [14]. The presence of undefined CD99 ligands on leukocyte surfaces as well as regulation of the immune response via interaction between CD99 and its ligands has been suggested [14]. Thus, identification of CD99 ligands has become necessary in order to broaden the understanding of CD99 functions involved in immunoregulation. The current study used recombinant CD99 and the pull-down method with DTSSP cross-linkers, as well as LC–MS/MS analysis, to identify CD99 ligands.

## Main text

### Methods

#### Large scale production of recombinant CD99HIgG Fc fusion proteins

HEK293T cells stably expressing CD99HIgG were generated in our laboratory [14]. Large scale production of recombinant CD99HIgG was performed as described in [14]. To determine the purity, purified CD99HIgG was resolved in 10% SDS-PAGE. Purity of CD99HIgG was detected by staining SDS-PAGE gels with Coomassie blue. The structure of CD99HIgG was determined by western blotting using anti-CD99 mAb followed by rabbit anti-mouse immunoglobulins antibodies-HRP or anti-human immunoglobulins antibodies-HRP.

#### Immunofluorescence staining of CD99 ligands using DTSSP

To validate the binding activity of purified CD99HIgG, PBMCs were stained with 20 μg/mL of biotinylated CD99HIgG or CD147ExHIgG control as described in [14]. The anti-CD3 mAb-FITC (BD Biosciences, San Jose, CA, USA) and anti-CD14 mAb-PerCP (BioLegend, San Diego, CA, USA) were used for determining cell subpopulations and analysed using a BD Accuri C6 flow cytometer (BD Biosciences).

To study the cellular distribution of CD99 ligands, Fc receptors of various human hematopoietic cell lines were blocked using 20% FBS-PBS-0.02% NaN_3_. Cells were stained with 10 μg/mL of CD99HIgG or CD147ExHIgG control on ice for 1 h. Next, 2 mM DTSSP (3,3′-dithiobis (sulfosuccinimidyl propionate); Pierce) was added, followed by further incubation for 2 h. Then, DTSSP was neutralized by adding 20% FBS and the un-crosslinked proteins were washed out. The bound proteins was determined using rabbit anti-human immunoglobulins antibodies-FITC and analysed via a FACSort flow cytometer (BD Biosciences).

#### Isolation of membrane bound CD99 ligands by pull-down method

THP-1 cells (1 × 10^8^) were stained with 10 μg of CD99HIgG or CD147ExHIgG control in 500 μl of PBS and incubated at 4˚C for 3 h. Next, an equal volume of 4 mM DTSSP was added, and further incubated for 2 h. Subsequently, 50 mM glycine in PBS was added at a final concentration of 20 mM. Cells were washed, then lysed using 1 mL of 1% lauryl maltoside containing protease inhibitors. Recombinant proteins-ligand complexes were isolated from cell lysate using protein G agarose beads (Pierce), eluted via 0.5% sodium dodecyl sulphate (SDS), and further investigated using western blotting. Molecular weight shifts of the complex were detected using anti-CD99 mAb or anti-CD147 mAb followed by rabbit anti-mouse immunoglobulins antibodies-HRP. This complex was subjected to LC–MS/MS.

#### Identification of CD99 ligands by LC–MS/MS

For sample preparation and LC–MS/MS analysis, the complex of CD99HIgG and its ligands as well as control proteins were suspended in 0.5% SDS. The samples were heated at 95 ˚C for 5 min and resolved via a 4% SDS-PAGE stacking gel at 20 mA for 25 min. Protein bands were developed using Coomassie Brilliant Blue R-250 dye. After de-staining, the developed bands were excised for in-gel digestion [see Additional file 1]. In addition, complexes of CD99HIgG and its ligands were visualized via western blotting on PVDF membranes, using anti-CD99 mAb followed by rabbit anti-mouse immunoglobulins antibodies-HRP. Protein bands were excised and subjected to on-membrane digestion [see Additional file 1].

## Results

### Recombinant CD99HIgG fusion proteins were produced

To identify CD99 ligands expressed on cell surfaces, the CD99HIgG fusion protein was produced in HEK293T cells and purified according to a previously described method [14]. Based on the method described [14], the purity of CD99HIgG was determined using SDS-PAGE, and its structure was confirmed via western blotting [see Additional file 2]. The results indicated that CD99HIgG was successfully produced. We then verified whether the CD99HIgG would bind to its ligand expressed on the cell surface. As predicted, the generated CD99HIgG bound to monocytes and CD3- lymphocyte sub-populations, but not to CD3 + T lymphocytes [see Additional file 3].

Considered together, the produced CD99HIgG was suitable for use in the identification of CD99 ligands expressed on cell surface.

### The putative CD99 ligands were expressed on human hematopoietic cell lines

Various human hematopoietic cell lines were stained using CD99HIgG. Of the tested cell lines, the THP-1 monocytic cell line and K562 erythroid cell line were positive for CD99HIgG staining [see Additional file 4], whereas, the recombinant CD99 protein bound weakly to the U937 monocytic cell line, the HL-60 myeloid cell line, and the SUP-T1 T cell line. No CD99 binding was observed in the KG1a myeloid cell line or other T cell lines. Based on these screening results, THP-1 cells were selected for further studies aimed at identifying the CD99 ligand.

### CD99 ligands were pulled down and identified via LC–MS/MS

**Fig. 1:**
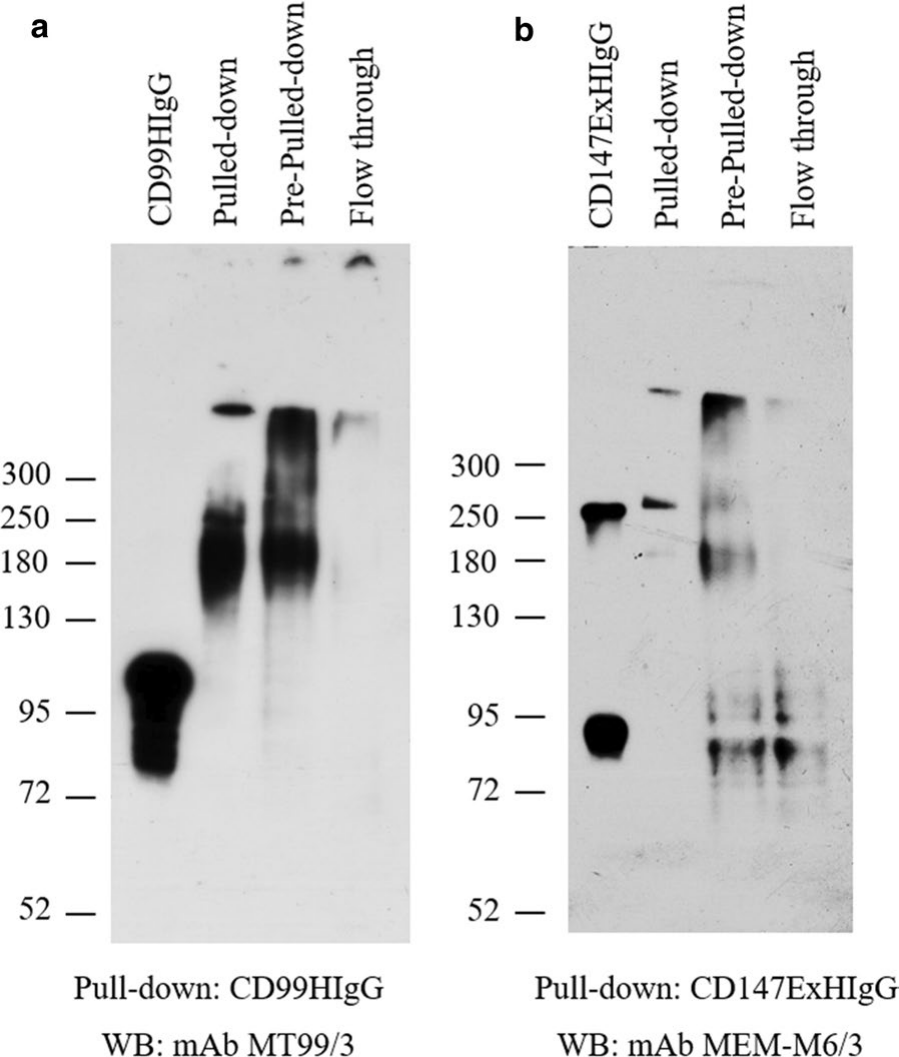
Identification of CD99 ligands by pull-down method with DTSSP cross-linker. THP-1 cells were stained with CD99HIgG or CD147ExHIgG fusion proteins and crosslinked with DTSSP. Following cell lysis, the complexes of CD99HIgG with their ligands were pulled-down using protein G beads. **a** CD99HIgG or **b** CD147ExHIgG was used as bait protein for pull-down experiments. Western blotting under non-reducing conditions was performed using anti-CD99 mAb (MT99/3) or anti-CD147 mAb (MEM-M6/3), followed by HRP conjugated rabbit anti-mouse immunoglobulins antibodies. Lane 1; CD99HIgG or CD147ExHIgG recombinant proteins used in the pull-down method. Lane 2; pull-down material obtained via Protein A beads. Lane 3; pre-pull-down represents the material before the pull-down process. Lane 4; flow through represents the material obtained following the pull-down process. The molecular weights (kDa) of protein markers are displayed on the left

THP-1 cells, on which the CD99 ligand was strongly expressed, were used to pull-down CD99 ligands, utilizing CD99HIgG, as the bait protein, and a DTSSP cross-linker. The pulled-down materials were verified via western blotting. The shift in molecular weight (compared to purified CD99HIgG in lane 1) that was seen in the pre-pull-down lysate (lane 3) was not seen in the flow though lysate (lane 4); (Fig. 1a). The presence of pull-down complexes was confirmed by analysing pulled-down proteins in lane 2 (Fig. 1a). A complex between CD147ExHIgG control and any of the surface proteins was hardly observed (Fig. 1b). These results indicated that CD99 ligand-CD99HIgG complexes could be pulled-down from the surfaces of THP-1 cells.

In order to identify possible CD99 ligands, LC–MS/MS were performed. The CD99 ligand-CD99HIgG complexes obtained by way of the pull-down method were prepared for LC–MS/MS analysis via both in-gel and on-membrane digestion.

For in-gel digestion, pulled-down samples were resolved via SDS-PAGE. Protein bands were excised and processed by tryptic in-gel digestion. Purified CD99HIgG and bovine serum albumin (BSA) were used as controls in LC–MS/MS analysis. The peptide finger prints obtained via LC–MS/MS were searched for batch protein sequences in the NCBI database. Protein identification of BSA control showed that most proteins were bovine serum albumin [see Additional file 5]. Immunoglobulins, which formed part of the purified CD99HIgG control, were also identified [see Additional file 6]. The HIgG Fc region of CD99HIgG, the Fc receptor, and other candidate proteins in CD99 ligand-CD99HIgG complexes were identified (Table 1). Interestingly, several unnamed protein products and some surface receptors identified via high MASCOT protein scores were supposed CD99 ligands. In addition, the heat shock cognate 71 kDa protein, HSPA8/HSC70, was highly ranked. HSC70, an intracellular protein that may be associated with CD99 ligands, was pulled down with the CD99 recombinant protein.

**Table 1:** List of proteins obtained from in-gel digestion of CD99 ligands-CD99HIgG complexes followed by LC–MS/MS analysis and identified by MASCOT

Accession no	Mass	Protein score	Description
gi|28336	42128	248	Mutant beta-actin (beta ~ -actin) [Homo sapiens]
gi|119612723	22791	203	Actin, alpha, cardiac muscle, isoform CRA_b [Homo sapiens]
gi|7331218	66149	210	Keratin 1 [Homo sapiens]
gi|386854	52928	140	Type II keratin subunit protein, partial [Homo sapiens]
gi|114644568	60266	36	PREDICTED: keratin, type II cytoskeletal 6A [Pan troglodytes]
gi|553734	2269	104	Putative, T cell receptor alpha [Homo sapiens]
gi|24234686	53598	101	Heat shock cognate 71 kDa protein isoform 2 [Homo sapiens]
gi|194390750	23238	99	Unnamed protein product [Homo sapiens]
gi|48257068	64804	74	HSPA8 protein, partial [Homo sapiens]; Alternative name: heat shock cognate 71 kDa protein
gi|51095055	25582	72	Ser (D206s) [Homo sapiens]; Alternative name: heat shock cognate 71 kDa protein
gi|194384180	55158	71	Unnamed protein product, heat shock cognate 71 kDa protein [Homo sapiens]
gi|158257566	45481	25	Unnamed protein product, heat shock cognate 71 kDa protein [Homo sapiens]
gi|12653415	73967	88	Heat shock 70 kDa protein 9 (mortalin) [Homo sapiens]
gi|119582532	50291	78	Heat shock 70 kDa protein 9B (mortalin-2), isoform CRA_a [Homo sapiens]
gi|221041984	70193	74	Unnamed protein product [Homo sapiens]
gi|86651611	19208	27	Heat shock 70 kDa protein 9B [Homo sapiens]
gi|31,332	42978	57	Unnamed protein product, IgG Fc receptor [Homo sapiens] [Homo sapiens]
gi|180162	43253	42	Fc gamma receptor type I [Homo sapiens]
gi|28317	59720	54	Unnamed protein product, keratin [Homo sapiens]
gi|10337581	47325	47	Keratin, type I cuticular Ha3-II [Homo sapiens]
gi|4758344	9775	54	High affinity immunoglobulin epsilon receptor subunit gamma precursor [Homo sapiens]
gi|41059927	51928	48	Anti-HBs antibody heavy chain [Homo sapiens]
gi|401817557	24160	43	Chain A, Human IgG1 Fc Fragment [Homo sapiens
gi|10334541	42301	30	Immunoglobulin heavy chain [Homo sapiens]
gi|1890020	25913	48	Mutant keratin 9 [Homo sapiens]
gi|62896589	50435	43	Eukaryotic translation elongation factor 1 alpha 1 variant [Homo sapiens]
gi|62088522	21755	35	Ribosomal protein L12 variant [Homo sapiens]
gi|10800144	13928	34	Histone cluster 1, H2aj [Homo sapiens]
gi|4506691	16549	34	40S ribosomal protein S16 isoform 1 [Homo sapiens]
gi|519672190	13093	32	Immunoglobulin A heavy chain variable region, partial [Homo sapiens]
gi|37492	50810	31	Alpha-tubulin [Homo sapiens]
gi|5453595	5926	31	Adenylyl cyclase-associated protein 1 [Homo sapiens]
gi|119595845	193540	29	hCG1642754, isoform CRA_b [Homo sapiens]
gi|119627826	66420	28	Splicing factor proline/glutamine-rich (polypyrimidine tract binding protein associated), isoform CRA_a [Homo sapiens]
gi|1381146	66846	27	Hs-CUL-3, partial [Homo sapiens]
gi|693933	47421	21	2-phosphopyruvate-hydratase alpha-enolase [Homo sapiens]
gi|338215	54169	17	Activin type I receptor [Homo sapiens]

For the purpose of on-membrane digestion following electroblotting, protein bands corresponding to CD99 ligand-CD99HIgG complexes on the PVDF membrane were subjected to an on-membrane digestion protocol prior to LC–MS/MS analysis (Fig. 1a); the results are shown in Table 2. Although the number of proteins identified by this technique was less than that identified by the in-gel digestion technique, some unnamed protein products and surface receptors were observed.

**Table 2:** List of proteins obtained from on-membrane digestion of CD99 ligands-CD99HIgG complexes followed by LC–MS/MS analysis and identified by MASCOT

Accession no	Mass	Protein score	Description
gi|189054178	66151	47	Unnamed protein product [Homo sapiens]
gi|375314779	66197	33	Keratin 1 [Homo sapiens]
gi|119617032	60159	30	Keratin 6B, isoform CRA_a [Homo sapiens]
gi|33338088	7250	27	MSTP132 [Homo sapiens]
gi|1326083	59537	22	Butyrophilin precursor [Homo sapiens]
gi|16075980	8251	20	Immunoglobulin lambda chain variable region [Homo sapiens]
gi|119619622	42404	20	Reticulocalbin 2, EF-hand calcium binding domain [Homo sapiens]
gi|17939630	48259	18	Hepatocyte nuclear factor 3, beta, partial [Homo sapiens]
gi|987870	68485	18	RNase L inhibitor [Homo sapiens]
gi|339303	12211	18	T-cell receptor alpha, partial [Homo sapiens]
gi|34526665	16528	16	Unnamed protein product [Homo sapiens]
gi|28317373	24683	16	TPA: IL-1F7b (IL-1H4, IL-1H, IL-1RP1) [Homo sapiens]
gi|10438579	76494	16	Unnamed protein product [Homo sapiens]
gi|402230	5045	16	T-cell receptor beta chain [Homo sapiens]
gi|155722985	129980	15	Structural maintenance of chromosomes protein 5 [Homo sapiens]
gi|5834152	13979	15	Immunoglobulin heavy chain variable region [Homo sapiens]
gi|21740017	90368	15	Hypothetical protein [Homo sapiens]
gi|4504779	88655	15	Integrin beta-8 precursor [Homo sapiens]
gi|5453932	13342	14	DNA-directed RNA polymerase II subunit RPB11-a [Homo sapiens]

Possible CD99 ligands of interest, as indicated by the results of both in-gel and on membrane digestions, are listed to facilitate identification of CD99 ligands.

## Discussion

CD99 plays the vital role in immunoregulation, with particular reference to T cell regulation. However, the precise function of CD99 in T cell regulation remains unclear. As a result, CD99 ligand remains undefined. Therefore, identification of the CD99 ligand is necessary. Interestingly, as our previously reported, the putative CD99 ligand is a membrane protein. It was found that CD99HIgG with a DTSSP cross-linker bound specifically to the surfaces of monocytes, NK cells and dendritic cells, but not to those of B and T cells [14]. In the current study, we produced a new batch of CD99HIgG from a stable clone of HEK293T cells. Its structure was similar to that described in our former publication [14]. CD99HIgG is composed of the extracellular region of CD99 linked to the hinge region and Fc region of HIgG1. It is secreted as a dimeric form with a molecular weight of 100 kDa. In regard to its binding activity, it binds to monocytes and CD3- lymphocyte subpopulations, but not to CD3+ T cells. Hence, the produced CD99HIgG were used to search for CD99 ligand expression on various hematopoietic cell lines by an immunofluorescence technique that uses DTSSP as cross-linker [15, 16]. The results demonstrated that the THP-1 monocytic cell line and the K562 erythroid cell line were markedly positive for CD99HIgG staining. The results obtained from both PBMC and hematopoietic cell lines indicated that CD99 ligands were strongly expressed on peripheral monocytes, which are related to the THP-1. CD99 ligands were not observed on the surface of CD3+ T cells as well as on all tested T cell lines, with the exception of SUP-T1 which showed a weak expression. We suggest that CD99 ligands expressed on antigen presenting cells are capable of binding with CD99 molecules that are highly expressed on T cells.

The pull-down method, which involves recombinant CD99 protein and a DTSSP cross-linker, was utilized to identify the amino acid sequences of CD99 ligands. THP-1 cells were used to represent CD99 ligand expressing cells. CD99HIgG was used as a bait protein and DTSSP was added to crosslink recombinant CD99 and its ligands on THP-1 cell surface. According to the properties of DTSSP, molecular weight shifts due to crosslinking between CD99 and CD99 ligands was observed in the pull-downs by western blotting under non-reducing conditions. The results indicated that recombinant CD99 was able to pull-down CD99 ligands from the surface of THP-1 cells. This is the first experiment that concretely demonstrated the presence of membrane CD99 ligand proteins. The samples obtained by the pull-down method were prepared for identification of CD99 ligands via LC–MS/MS.

Two methods, in-gel digestion and on-membrane digestion, were utilized to prepare samples for LC–MS/MS analysis. Several unnamed proteins and some surface receptors were obtained. These proteins were potential CD99 ligands. The unnamed protein products, in particular, should be the focus of attention, as no CD99 ligand has been identified so far. We speculated that the CD99 ligand may be a novel, un-defined protein. The possibility of heterophilic interaction of CD99 is suggested.

To the best of our knowledge, our experiments, are the first to confirm the presence of putative CD99 ligands on the surfaces of leukocytes. Several proteins that had been predicted to be CD99 ligand candidates were also included in the results of this study.

## Limitations

CD99 ligand candidates identified by LC–MS/MS have not yet been further confirmed. Additional investigations using different method i.e. Genome-scale receptor array (GSRA) technology are required to clarify CD99 ligands, in order to fully comprehend the role of CD99 in immunoregulation. Several specific antibodies to un-defined proteins are also required for confirming the CD99 ligand.

## Supplementary information

**Additional file 1**. Methods for identification of CD99 ligands by LC-MS/MS.

**Additional file 2: Figure S1**. Validation of the purity and structure of purified CD99HIgG.

**Additional file 3: Figure S2.** Verification the binding activity of purified CD99HIgG.

**Additional file 4: Figure S3**. The expression of CD99 ligands on hematopoietic cells.

**Additional file 5: Table S1**. List of proteins obtained from in-gel digestion of the BSA protein control followed by LC–MS/MS analysis and identified by MASCOT.

**Additional file 6: Table S2.** List of proteins obtained from in-gel digestion of purified CD99HIgG protein control followed by LC–MS/MS analysis and identified by MASCOT.

## Data Availability

All data generated or analysed during this study are included in this published article [and its Additional files 1, 2, 3, 4, 5, 6].

## Abbreviations

BSA: Bovine serum albumin
CD: Cluster of differentiation
mAbs: Monoclonal antibodies
