# Supplementary material for: The presence of membrane bound CD99 ligands on leukocyte surface
Source: BMC Res Notes. 2020 Oct 22;13:496. doi: 10.1186/s13104-020-05347-0 (PMC7583281; doi:10.1186/s13104-020-05347-0)
Supplement: Supplementary file 1 — Additional file 1. Methods for identification of CD99 ligands by LC-MS/MS. [file 13104_2020_5347_MOESM1_ESM.docx]

**Additional file 1**

**Methods for identification of CD99 ligands by LC-MS/MS**

For the in-gel digestion method, bands of interest that formed on the 4% stacking gel were excised (approximately 1×1 mm^2^ sections). Gels were destained and the proteins were washed using 50% methanol containing 50 mM ammonium bicarbonate. Clear gel plugs were dehydrated in 200 μl of acetonitrile and incubated at room temperature for 5 min. After incubation, acetonitrile was discarded and gels were further dried at room temperature. Disulphide bonds were reduced by adding of 10 mM dithiothreitol (DTT) in 10 mM ammonium bicarbonate and incubating at 56˚C for 1 h. After the supernatant was discarded, proteins were alkylated with 100 mM iodoacetamide (IAA) in 10 mM ammonium bicarbonate and incubated at room temperature in the dark for 1 h. Following the removal of the supernatant, gels were dehydrated using 200 μl of acetonitrile and incubated at room temperature for 5 min and dried again. Proteins were digested by adding 200 ng of trypsin in 10 mM ammonium bicarbonate and incubating overnight at 37˚C. Supernatant containing peptide was collected and dried overnight at 40˚C. Pellets were suspended with 20 μl of 0.1% formic acid for LC-MS/MS analysis.

For the on-membrane digestion method, protein bands (around 1×1 mm^2^) that formed on the PVDF membrane were excised. The membranes were washed thrice with distilled water. Then, 10 mM dithiothreitol (DTT) in 10 mM ammonium bicarbonate was added and incubated at 56˚C for 1 h. The supernatant was removed and 100 mM iodoacetamide (IAA) in 10 mM ammonium bicarbonate was added. After being incubated at room temperature in the dark for 1 h, the supernatant was discarded and 200 ng of trypsin in 10 mM ammonium bicarbonate was used to digest proteins. Following overnight incubation at 37˚C, the supernatant, containing peptides was collected and dried overnight at 40˚C. Pellets were suspended with 20 μl of 0.1% formic acid for LC-MS/MS analysis.

Digested peptide solutions were analysed via an Impact II UHR-TOF MS System (Bruker Daltonics Ltd., Germany) coupled to a nanoLC system: UltiMate 3000 LC System (Thermo Fisher Scientific, USA). Peptides were separated on a nanocolumn (PepSwift monolithic column 100 µm i.d. x 50 mm). Eluent A was 0.1% formic acid and eluent B was 80% acetonitrile in water containing 0.1% formic acid. Peptide separation was achieved via a linear gradient of 10% to 45% B for 8.5 min at a flow rate of 1 µl/min, including a regeneration step at 90% B and an equilibration step at 1% B; each run lasted 20 min. Peptide fragment mass spectra were acquired in the data-dependent AutoMS(2) mode, selecting most abundant precursor ions in 3 second cycles for fragmentation. The mass range of the MS scan was set from 150 to 2200 *m*/*z.* MS/MS data were submitted for a database search using the MASCOT search engine (http://www.matrixscience.com). For the purpose of protein identification, data were searched against the NCBI database. Search parameters were set as follows: taxonomy (*Homo sapiens*); enzyme (trypsin); variable modifications (carbamidomethyl, oxidation of methionine residues); mass values (monoisotopic); protein mass (unrestricted); peptide mass tolerance (1.2 Da); fragment mass tolerance (±0.6 Da), peptide charge state (1+, 2+ and 3+), Instrument (ESI-QUAD-TOF) and max missed cleavages (3). Ion score was −10×log (P), where P was the probability that observe a random matching event. Individual ions scores indicated identity or extensive homology (p < 0.05; a 1 in 20 chance of being a false positive). The protein score is the sum of the highest ions score for each distinct sequence.
