# Supplementary material for: The presence of membrane bound CD99 ligands on leukocyte surface
Source: BMC Res Notes. 2020 Oct 22;13:496. doi: 10.1186/s13104-020-05347-0 (PMC7583281; doi:10.1186/s13104-020-05347-0)
Supplement: Supplementary file 2 — Additional file 2: Figure S1. Validation of the purity and structure of purified CD99HIgG. [file 13104_2020_5347_MOESM2_ESM.docx]

**Additional file 2**


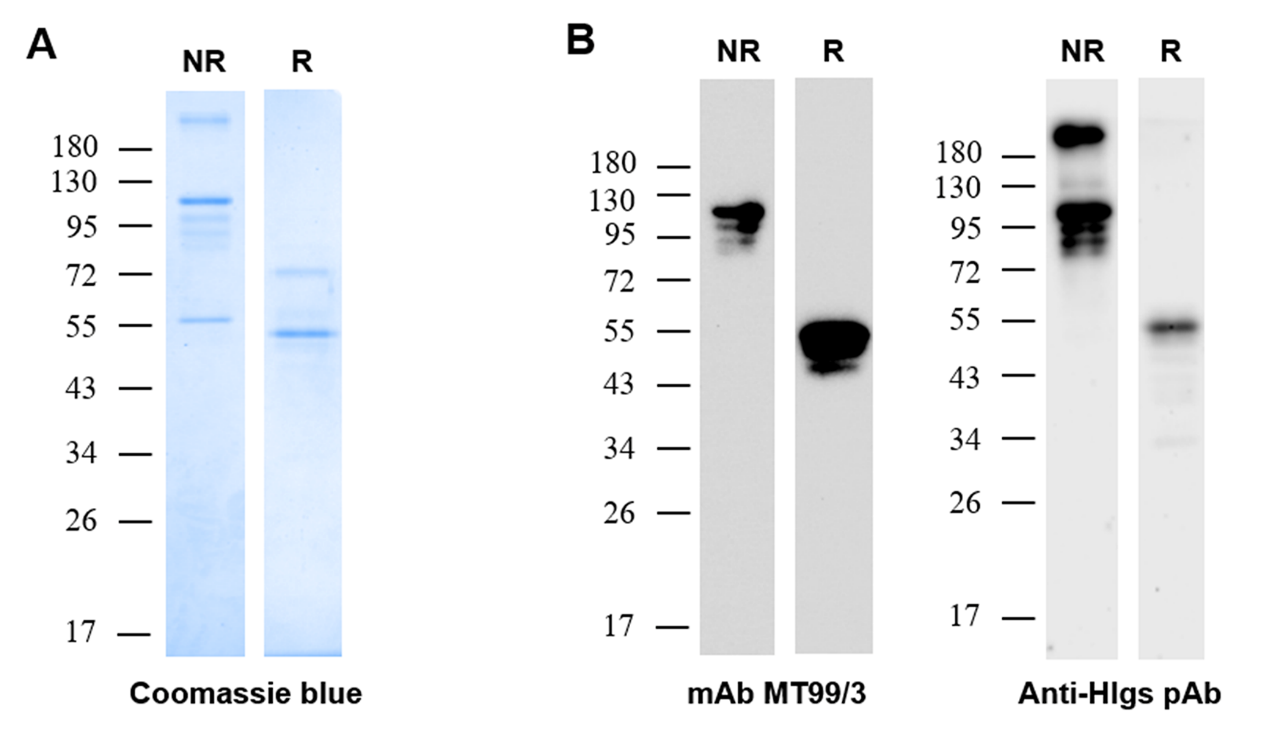


**Figure S1 Validation of the purity and structure of purified CD99HIgG.** Purified CD99HIgG was resolved in 10% SDS-PAGE under non-reducing (NR) and reducing conditions (R). (A) Protein bands were stained with Coomassie blue. (B) Western blotting was performed using anti-CD99 mAb (MT99/3) followed by HRP conjugated rabbit anti-mouse immunoglobulins antibodies or HRP conjugated anti-human immunoglobulins antibodies (anti-HIgs pAb). Protein markers with molecular weight (kDa) are shown on the left.
