# Supplementary material for: The presence of membrane bound CD99 ligands on leukocyte surface
Source: BMC Res Notes. 2020 Oct 22;13:496. doi: 10.1186/s13104-020-05347-0 (PMC7583281; doi:10.1186/s13104-020-05347-0)
Supplement: Supplementary file 3 — Additional file 3: Figure S2. Verification the binding activity of purified CD99HIgG. [file 13104_2020_5347_MOESM3_ESM.docx]

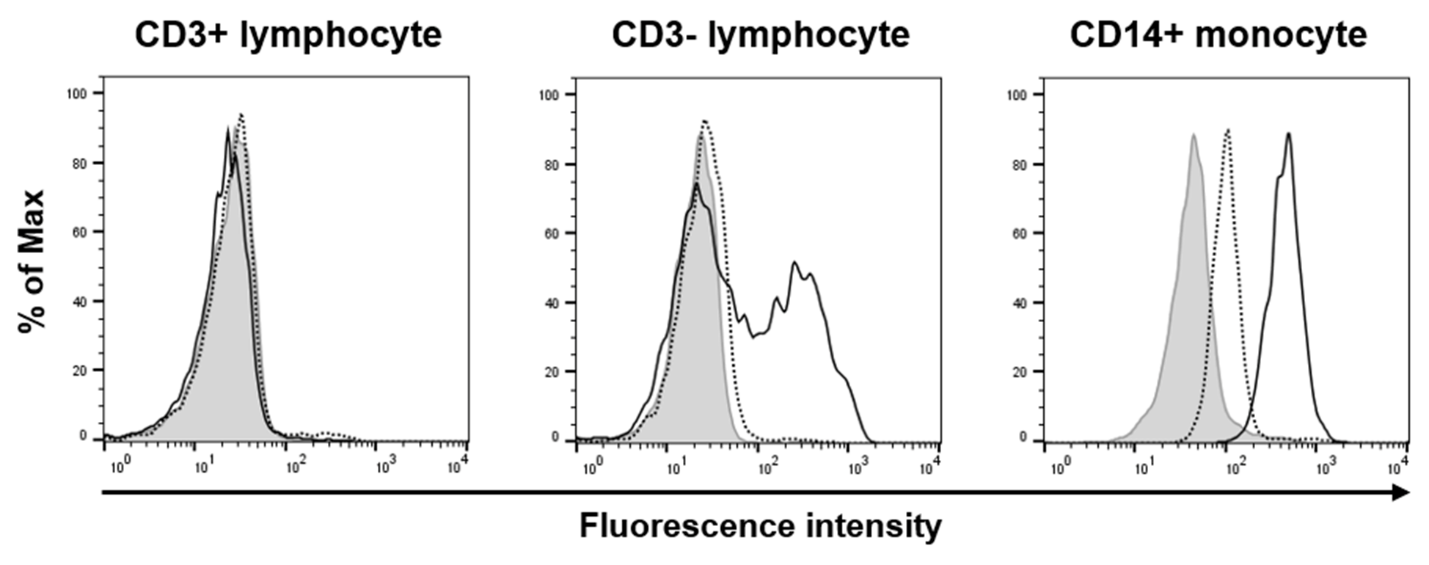
**Additional file 3**

**Figure S2 Verification the binding activity of purified CD99HIgG.** PBMCs were stained using biotin-labelled CD99HIgG or CD147ExHIgG control in combination with a DTSSP cross-linker. Streptavidin-PE was utilized to detect the binding of biotinylated recombinant proteins. Anti-CD3-FITC and anti-CD14-PerCP were used to determine indicated cell populations. No protein staining (grey peak), CD147ExHIgG (dotted line) and CD99HIgG (solid line).
