# Supplementary material for: The presence of membrane bound CD99 ligands on leukocyte surface
Source: BMC Res Notes. 2020 Oct 22;13:496. doi: 10.1186/s13104-020-05347-0 (PMC7583281; doi:10.1186/s13104-020-05347-0)
Supplement: Supplementary file 4 — Additional file 4: Figure S3. The expression of CD99 ligands on hematopoietic cells. [file 13104_2020_5347_MOESM4_ESM.docx]

**Additional file**
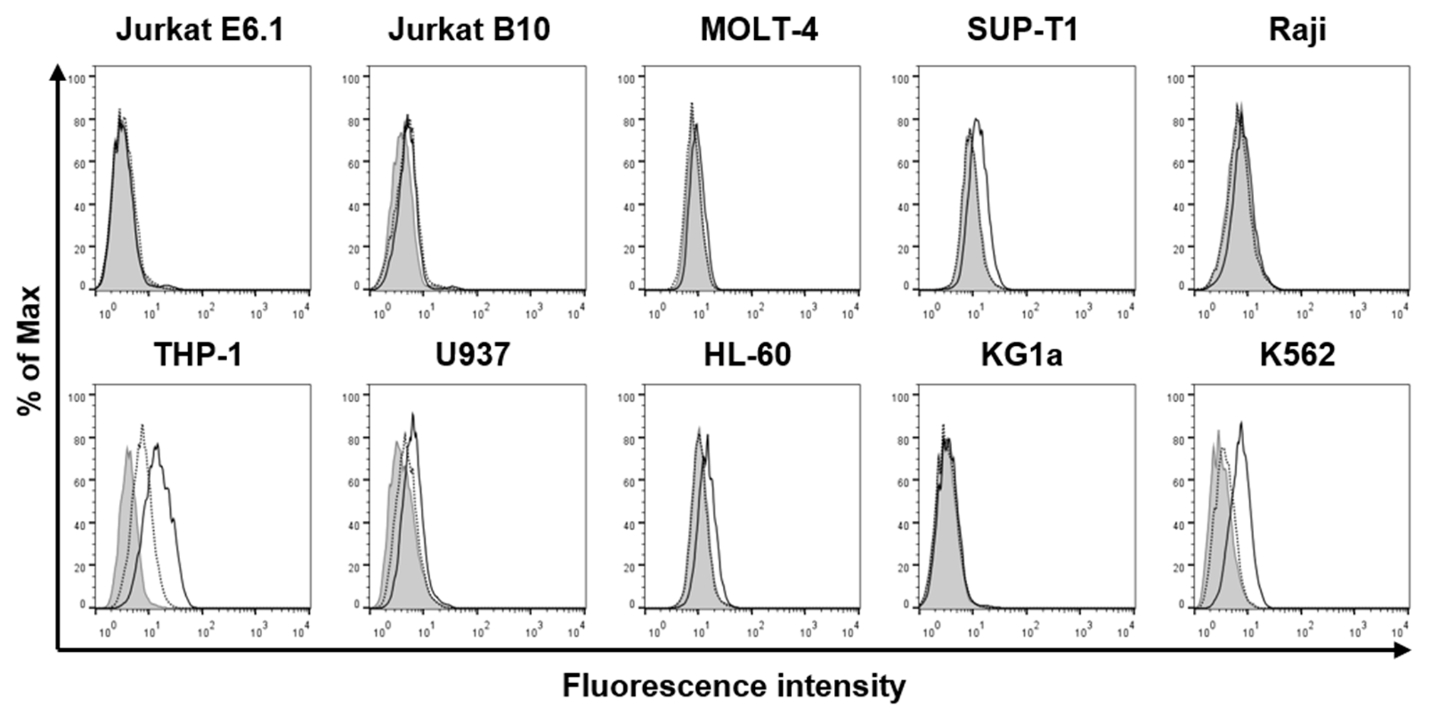
**4**

**Figure S3 The expression of CD99 ligands on hematopoietic cells.** Cell lines were stained with CD99HIgG or CD147ExHIgG control and a DTSSP cross-linker. Bound recombinant proteins were detected via FITC conjugated rabbit anti-human immunoglobulins antibodies. The overlay histograms of indicated cell types are shown. No protein staining (grey peak), CD147ExHIgG (dotted line) and CD99HIgG (solid line).
