# Supplementary material for: The presence of membrane bound CD99 ligands on leukocyte surface
Source: BMC Res Notes. 2020 Oct 22;13:496. doi: 10.1186/s13104-020-05347-0 (PMC7583281; doi:10.1186/s13104-020-05347-0)
Supplement: Supplementary file 5 — Additional file 5: Table S1. List of proteins obtained from in-gel digestion of the BSA protein control followed by LC–MS/MS analysis and identified by MASCOT. [file 13104_2020_5347_MOESM5_ESM.docx]

**Additional file 5**

**Table S1 List of proteins obtained from in-gel digestion of the BSA protein control followed by LC-MS/MS analysis and identified by MASCOT**

| **Accession no.** | **Mass** | **Protein score** | **Description** |
| --- | --- | --- | --- |
| ALBU_BOVIN  ALBU_MOUSE  ALBU_SHEEP  ALBU_FELCA  ALBU_PIG  ALBU_CANLF  ALBU_MESAU  ALBU_MACFA  ALBU_HUMAN  ALBU_CAPHI  ALBU_RABIT  ALBU_HORSE  FETA_BOVIN  ALBU_EQUAS  ALBU_RAT | 71244  70700  71139  70611  71643  70556  70177  70825  71317  10048  70861  70550  70368  70490  70682 | 4709  1188  1132  1573  1285  1204  1143  476  442  160  107  79  47  46  29 | Serum albumin OS=Bos taurus  Serum albumin OS=Mus musculus  Serum albumin OS=Ovis aries  Serum albumin OS=Felis catus  Serum albumin OS=Sus scrofa  Serum albumin OS=Canis lupus familiaris  Serum albumin OS=Mesocricetus auratus  Serum albumin OS=Macaca fascicularis  Serum albumin OS=Homo sapiens  Serum albumin OS=Capra hircus  Serum albumin OS=Oryctolagus cuniculus  Serum albumin OS=Equus caballus  Alpha-fetoprotein OS=Bos Taurus  Serum albumin OS=Equus asinus  Serum albumin OS=Rattus norvegicus |
