# Supplementary material for: The presence of membrane bound CD99 ligands on leukocyte surface
Source: BMC Res Notes. 2020 Oct 22;13:496. doi: 10.1186/s13104-020-05347-0 (PMC7583281; doi:10.1186/s13104-020-05347-0)
Supplement: Supplementary file 6 — Additional file 6: Table S2. List of proteins obtained from in-gel digestion of purified CD99HIgG protein control followed by LC–MS/MS analysis and identified by MASCOT. [file 13104_2020_5347_MOESM6_ESM.docx]

**Additional file 6**

**Table S2 List of proteins obtained from in-gel digestion of purified CD99HIgG protein control followed by LC-MS/MS analysis and identified by MASCOT**

| **Accession no.** | **Mass** | **Protein score** | **Description** |
| --- | --- | --- | --- |
| gi\|10799664  gi\|34535785  gi\|243169  gi\|2414492  gi\|218512079  gi\|10334551  gi\|223060  gi\|444737661  gi\|12054078  gi\|222995  gi\|34365089  gi\|386789  gi\|226337  gi\|13529281  gi\|7331218  gi\|194380064  gi\|7160995  gi\|323433100  gi\|553788  gi\|119614117  gi\|115511036  gi\|33871957  gi\|328942858  gi\|767884147  gi\|7657236  gi\|31542731 | 36525  60769  39383  26076  36505  42273  12910  27457  36662  12928  53071  52254  13452  29068  66149  125111  11532  15939  55207  24971  203428  16762  11771  34955  31757  59287 | 289  269  228  211  209  208  188  166  150  147  130  107  61  33  41  34  32  32  31  30  27  26  24  24  22  21 | immunoglobulin heavy chain constant region  [Homo sapiens]  unnamed protein product [Homo sapiens]  Ig gamma 2 H chain, BUR [human, Peptide Mutant, 348 aa]  immunoglobulin heavy chain, constant region [Homo sapiens]  RecName: Full=Ig gamma-2 chain C region  immunoglobulin heavy chain [Homo sapiens]  IgG2 pFc~ PIG Gm  immunoglobulin light chain lambda, partial [Homo sapiens]  immunoglobulin heavy chain constant region gamma 4 [Homo sapiens]  IgG3m(g) pFc HER  hypothetical protein [Homo sapiens]  hemopexin precursor, partial [Homo sapiens]  hemopexin  HPX protein [Homo sapiens]  keratin 1 [Homo sapiens]  unnamed protein product [Homo sapiens]  immunoglobulin heavy chain [Homo sapiens]  immunoglobulin variable region [Homo sapiens]  transferrin, partial [Homo sapiens]  twist homolog 1 (acrocephalosyndactyly 3; Saethre-Chotzen syndrome) (Drosophila) [Homo sapiens]  alpha-protein kinase 3 [Homo sapiens]  Glutaredoxin 5 [Homo sapiens]  immunoglobulin heavy chain variable region [Homo sapiens]  PREDICTED: mucin-1-like [Homo sapiens]  inositol monophosphatase 2 [Homo sapiens]  major facilitator superfamily domain-containing protein 7 isoform 2 [Homo sapiens] |
